# Nanotechnology—A Light of Hope for Combating Antibiotic Resistance

**DOI:** 10.3390/microorganisms11061489

**Published:** 2023-06-03

**Authors:** Ghazala Muteeb

**Affiliations:** Department of Nursing, College of Applied Medical Science, King Faisal University, Al-Ahsa 31982, Saudi Arabia; graza@kfu.edu.sa

**Keywords:** antibiotics, antibiotics resistance, nanotechnology, heavy metals, global health

## Abstract

Antibiotic usage and resistance are major health concerns. Antibiotic resistance occurs when bacteria evolve to resist the effects of antibiotics, making it impossible to treat infections. The overuse and misuse of antibiotics are the main contributing factors, while environmental stress (such as heavy metals accumulation), unhygienic conditions, illiteracy, and unawareness also contribute to antibiotic resistance. The slow and costly development of new antibiotics has lagged behind the emergence of antibiotic-resistant bacteria, and the overuse of antibiotics leads to negative consequences. The current study used different literature resources to generate an opinion and find a possible solution to antibiotic barriers. Different scientific approaches have been reported to overcome antibiotic resistance. The most useful approach among these is nanotechnology. Nanoparticles can be engineered to disrupt bacterial cell walls or membranes, effectively eliminating resistant strains. Additionally, nanoscale devices enable the real-time monitoring of bacterial populations, allowing for the early detection of resistance emergence. Nanotechnology, along with evolutionary theory offers promising avenues in combating antibiotic resistance. Evolutionary theory helps us understand the mechanisms by which bacteria develop resistance, allowing us to anticipate and counteract their adaptive strategies. By studying the selective pressures that drive resistance, we can therefore design more effective interventions or traps. The synergy between the evolutionary theory and nanotechnology presents a powerful approach to combat antibiotic resistance, offering new avenues for the development of effective treatments and the preservation of our antibiotic arsenal.

## 1. Introduction

Antibiotic resistance is a significant public health issue that has the potential to impact millions of people worldwide [1]. Antibiotic resistance occurs when bacteria evolve to resist the effects of antibiotics, making it difficult or impossible to treat infections caused by these bacteria with standard antibiotic therapies [2]. Antibiotics (penicillin, tetracycline, ciprofloxacin etc.) are medications used to treat bacterial infections from acute to chronic [3], and are considered life-saving drugs. There are different generations of antibiotics, each with their distinct characteristics and uses. Currently, there are five generations discovered according to the variant of bacteria. The fifth-generation of antibiotics are the most recent that are used for treating Gram-positive resistant bacteria including MRSA (methicillin-resistant *Staphylococcus aureus*) and urinary tract infections (UTI) such as linezolid and daptomycin [4]. Therefore, antibiotic resistance has the potential to cause widespread health crises, as antibiotic-resistant bacteria can spread rapidly and cause infections that are difficult to treat. Inadequate healthcare infrastructure, such as limited access to healthcare services, poor infection control, limited diagnostic capabilities, and inappropriate antibiotic use, can all contribute to the emergence and spread of antibiotic resistance. Furthermore, antibiotic overuse, misuse, or incorrect prescriptions in healthcare settings can all lead to the selection and growth of resistant microorganisms. Inadequate sanitation and hygiene standards in healthcare institutions might also help resistant infections proliferate further [5]. Antibiotic resistance, on the other hand, can have a negative impact on health, particularly when effective drugs are restricted or ineffective against resistant infections. Antibiotic resistance makes it difficult to cure common diseases, which results in prolonged hospitalizations, greater rates of morbidity and mortality, and more expensive healthcare. Inadequate treatment options brought on by antibiotic resistance can put a burden on medical facilities and lower the standard of care given [6]. The use of antibiotics in animal husbandry and agriculture also contributes to antibiotic resistance development, as it increases the likelihood of exposure to antibiotic-resistant bacteria [7]. Landfill leachate and wastewater are also possible sources and reservoirs of drug-resistant microorganisms. These conditions can aid in the spread and persistence of antibiotic-resistant microorganisms. Landfills also accept a diverse spectrum of garbage, including pharmaceutical waste and wasted antibiotics. These compounds can seep into the environment and contaminate the groundwater or surface water, resulting in the presence of antibiotics and antibiotic-resistant microorganisms [8]. Similarly, water from numerous sources, including hospitals, households, and agricultural activities, contain a varied diversity of bacteria, including antibiotic-resistant forms. Wastewater treatment plants may not properly remove antibiotic residues or resistant microorganisms, allowing them to infiltrate the environment. Through irrigation with treated wastewater, effluent from these treatment plants can transmit antibiotic-resistant bacteria and resistance genes into the surface waters, soil, and even agricultural areas [9].

**Figure 1:**
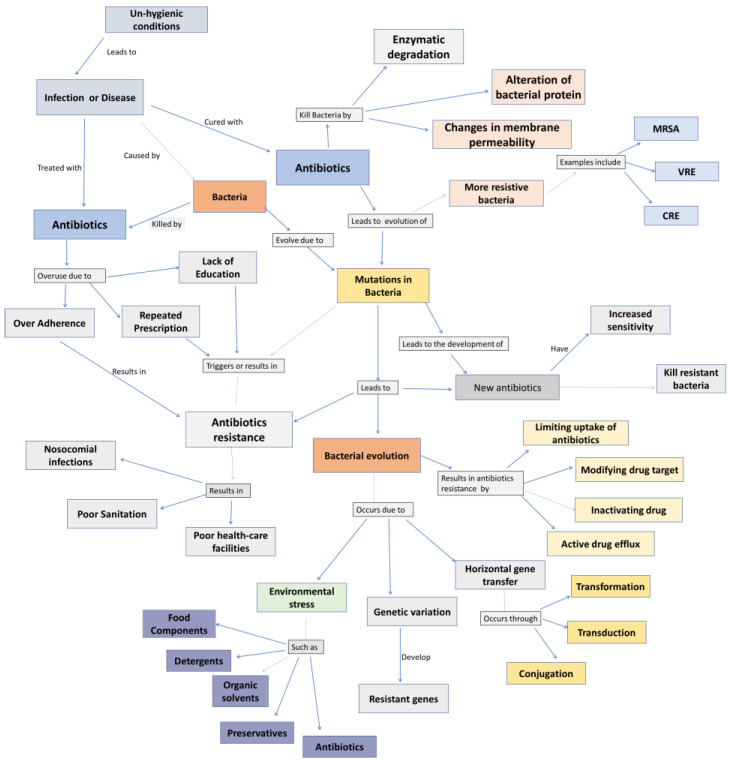
Summary of the complete antibiotic resistance phenomenon in the form of a flow chart.

The slow and expensive development of new antibiotics has lagged behind the emergence of antibiotic-resistant bacteria, and antibiotic overuse can have negative consequences such as the selection of antibiotic-resistant strains of non-pathogenic bacteria [10]. Therefore, considering these consequences and leading factors, the possible solutions reported by scientists are discussed in detail with evidence supporting their significance. Figure 1 summarizes the entire antibiotic resistance phenomenon.

In generating the current opinion paper, the focus was on collecting data from the past five years and utilizing case studies, research articles, and review articles as sources of information. The domain coverage employed artificial intelligence to assess antibiotic usage and resistance among patients. Possible solutions in the scientific world were explored to identify the most effective approach to combat antibiotic resistance, resulting in the identification of nanotechnology as the most powerful tool.

## 2. Viewpoint and Discussion with Conclusions

### 2.1. Antibiotic Resistance—A Menace to the Global Community

The use of antibiotics has brought about a significant change in the treatment of infectious diseases worldwide, playing a vital role in the increased life expectancy during the 20th century by reducing the mortality rates caused by infectious diseases [11]. Meanwhile, in different case studies, it has been investigated that globally, the consumption of antibiotics for human health has risen by 65% from 2000 to 2015, with the majority of the increase occurring in LMICs (low-to-middle income countries) [12]. In a cross-sectional study conducted at Kwame Nkrumah University of Science and Technology hospital in the Ashanti region of Ghana using electronic medical records (EMR), it was reported that the percentage of patient encounters resulting in antibiotic prescriptions was higher than the WHO’s recommended optimum of 27%, with approximately 33–36% of monthly encounters resulting in antibiotics being prescribed. Almost half of the prescribed antibiotics belong to the Watch group designated by the WHO. Among the most commonly prescribed antibiotics were amoxicillin-clavulanic acid (50%), azithromycin (29%), ciprofloxacin (28%), metronidazole (21%), and cefuroxime (20%) [13]. The LMICs face preventable deaths as a result of concurrent problems with antibiotic misuse and underuse, as well as the lack of regular access to antibiotics due to inadequate drug supply chains or prices in LMICs, as stated by Dr. Ramanan Laxminarayan in the report “Access Barriers to Antibiotics” from the CDDEP (Center for Disease Dynamics, Economics & Policy) [14]. To treat major disease, both viral and bacterial antibiotics are prescribed according to the diagnosed infection. The lack of patient records and antibiograms is a key driver of antibiotic resistance, leading to the inappropriate use of antibiotics worldwide, especially in LMICs. To address this issue, both doctors and consultants require proper guidelines and charts before assigning prescriptions to infectious patients. These guidelines should consider local resistance patterns and the patient’s medical history. A high level of follow-up on antibiograms should also be observed to ensure that patients receive effective antibiotic treatment. Monitoring antibiotic prescribing practices is critical to combat antibiotic resistance. The WHO has developed guidelines to help countries reduce the misuse and overuse of antibiotics. Countries should implement measures to promote the responsible use of antibiotics, such as public education campaigns and surveillance programs to track antibiotic resistance patterns. Promoting the development of new antibiotics is also essential to combat antibiotic resistance. Investment in antibiotic research and development is necessary to ensure that new antibiotics can be developed to treat emerging infectious diseases. Hence proper prescribing guidelines, monitoring antibiotic prescribing practices, and promoting the responsible use of antibiotics can thereby slow the evolution of antibiotic resistance and safeguard public health. 

### 2.2. Heavy Metals and Antibiotic Resistance

ARGs (antibiotic resistance genes) are a present environmental threat that can influence various environmental species and media [15]. ARGs pose a greater potential environmental risk than antibiotics and have a significant impact on human health [16]. Reports suggest that antibiotic resistance-related illnesses affect around two million people annually in the United States, with a mortality rate of 1.5%. On a global level, antibiotic-resistant infections cause the deaths of 700,000 people each year [17]. Antibiotic usage and heavy metals as feed additives cause the existence of ARGs in the soil environment. However, the impact of their presence on ARGs, microorganisms, and environmental factors is not well understood. A case study aimed at investigating the relationship among ARGs, environmental factors, mobile genetic elements (MGEs), and microorganisms by selecting sulfadiazine (Sd) and cadmium (Cd) as antibiotic substances and representative heavy metals, respectively revealed that the addition of Sd and Cd, in combination or separately, increased the total abundance of the MGEs and ARGs. The bacterial phyla Proteobacteria and Bacteroidetes were found to be the most prevalent in this study. The presence of mobile genetic elements (MGEs), particularly intI1, was found to have a significant influence on the abundance and dissemination of the ARGs. It is therefore crucial to conduct a full assessment of both the ecological and environmental risks of the appearance and development of ARGs in the soil environment as a consequence of its contamination with antibiotics and heavy metals [18]. In another case study, the status of heavy-metal pollution and drug-resistant *E. coli* characteristics in Shandong Province, China from swine-farm wastewater was investigated. Mineral elements were measured, and 29 antibiotic-resistant and resistance genes were evaluated in *E. coli* isolated from the wastewater. The findings revealed severe pollution from both zinc and iron, and a high degree of resistance in most *E. coli* isolates to chloramphenicol, tetracyclines, and sulfonamides. The clonal complex 10 was prevalent, and multidrug-resistant *E. coli* was found to be extensively distributed [19,20]. Meanwhile, several other studies suggested that the spread of antibiotic-resistant genes could be influenced by the contamination of heavy metals. Human activities that cause environmental pollution may result in mutagenesis due to the interaction between antibiotic effectiveness and the development of resistance mechanisms under stressful conditions. In addition, acquired resistance could involve specific efflux pumps that are encoded by plasmids. The case studies mentioned above provide compelling evidence in that environmental factors, particularly heavy metals, play a significant role in antibiotic resistance and its exacerbation. To effectively address the issue of antibiotic resistance, it is crucial to control pollution and employ significant measures to promote a healthy environment. The environmental factor in antibiotic resistance is considered as a primary factor in tackling the challenges posed by antibiotic resistance. By prioritizing environmental health and implementing effective pollution control measures, we can therefore reduce the prevalence of antibiotic resistance and safeguard public health. 

### 2.3. Nanotechnology—A Combatting Approach for Antibiotic Resistance

The discovery and development of antibiotics during the 20th century played a significant role in fighting bacterial infections. However, the constantly evolving bacterial genomes lead to a more serious issue of bacterial antibiotic resistance. To overcome this problem, nanotechnology is being employed across various fields, such as nanoparticle-enabled antibacterial vaccination and targeted antibiotic delivery [21]. Multiple cases have reported gold, silver, and zinc oxide nanoparticles to be effective antimicrobial agents against multidrug-resistant bacteria such as MRSA, *Pseudomonas*, *Klebsiella*, and *Escherichia coli.* The utilization of nanoparticles as carriers for antimicrobial agents offers advantages such as improved bioavailability, reduced likelihood of drug toxicity, and accumulation of sub-therapeutic drugs. Additionally, nano photothermal therapy employing the nanostructures of functionalized antibodies and fullerene represents another potential strategy that could prove to be beneficial [22]. However, despite their potential to help fight antibiotic-resistant bacteria, nanopreparations can in fact have ecotoxic qualities. Although they show promise for a number of uses, including antibacterial activity, it is crucial to take into account any potential negative impacts on the environment. Nanoparticles can be hazardous to organisms and the environment in varying degrees. Their small size, huge surface area, and distinctive physicochemical characteristics can cause interactions with ambient elements and biological things. These interactions can have a negative impact on species, ecosystems, and ecological processes. Comprehensive risk assessment procedures should therefore be established to address the possible ecotoxicity of nano preparations. This includes assessing the hazards and exposure pathways, as well as the possible environmental and human health consequences. Regulatory frameworks should be in place to guarantee that nanopreparations can be developed, used, and disposed of in an ethical and sustainable manner [23,24].

Numerous techniques employing nanotechnology-based drug delivery systems, including liposomes [25,26], polymeric nano-vehicles, [27] and nanoparticles of silica mesoporous [28], have been validated and deemed to be effective in the fight against drug-resistant bacterial strains. Considering all practical applications of nanoparticles, researchers are currently exploring potential drug delivery systems using nanoparticles to enhance the therapeutic use of antibiotics with minimized drawbacks. Examples of applied nanomaterials to combat drug resistance are represented in Table 1.

Meanwhile, nanotechnology strategies that can interfere with the biofilm formation (a major factor in bacterial resistance development against antibiotics) are reported to be the most effective approach [37]. Overcoming antibiotic resistance, especially due to biofilms, is a crucial factor for overcoming the challenges of antibiotic resistance in general. Biofilms are functioning bacterial clusters formed, among other things, due to the production of EPSs (extracellular polymeric substances) by these microorganisms. EPSs are the main cause of the ineffectiveness of therapies for biofilm infection, and limit permeability due to the insufficient accumulation of therapeutic drugs within the biofilm. The unique features of biofilm matrix barriers are due to the components of the biofilm that are charged negatively with the highly viscous and compact EPS structures. However, physical nanoparticles (degrading enzymes, nano-motors, or microneedle patches) can treat even the biofilm antibiotic resistance [38,39]. From all the above-mentioned case studies and reviews, it has been very clear that in the future, nanotechnology can be a promising approach for treating antibiotic resistance and ensure public health and safety. The treatment of bacteria at the nanoscale is the most appropriate method to deal with the current environmental stress and bacterial evolution. The mutations occurring in bacteria leading to the production of new resistant bacterial strains is a threat to humanity. Therefore, its fight at the nanoscale can stop the war at the macroscopic level.

Recent research has demonstrated that microorganisms can evolve resistance to both ionic and nano-metals. While nanomaterials have shown promise in treating drug resistance, it is vital to consider the possibility of microbial adaptability and the evolution of resistance mechanisms. Microbes have a variety of adaptation and survival tactics in the presence of antimicrobial agents, including metals. Metal resistance methods include efflux pumps, which actively remove metals from the cell, metal sequestration by intracellular binding proteins, and genetic mutations that change the metal-binding sites or metabolic pathways. Studies have shown that bacteria and other microbes can acquire resistance to nanometals such as copper nanoparticles, silver nanoparticles, and zinc oxide nanoparticles. This resistance can develop as a result of genetic alterations that provide protection against the harmful effects of these nanomaterials. It is important to note that the rate and level of resistance development might vary based on various parameters, including the individual microbe, the type and concentration of the nanomaterial, the exposure settings, and the period of exposure itself. Furthermore, the possibility of cross-resistance between the nanometals and traditional antibacterial treatments is an essential consideration. To address the issue of resistance development, researchers are investigating tactics such as combination therapy, targeted delivery systems, and the development of new nanomaterials with better antibacterial capabilities. Ongoing research strives to better understand the processes of resistance and identify solutions to lessen or eliminate it. To summarize, while nanomaterials have the potential to combat drug resistance, it is crucial to acknowledge the microorganisms’ ability to evolve resistance to both ionic and nano-metals [40,41,42].

Furthermore, it is important to recognize that assessing the efficiency of nanomaterials against microorganisms and the possibility for resistance development necessitates meticulous and well-designed research. Short-term testing periods of 24 or 48 h may not capture the whole evolution of resistant clones, nor provide a comprehensive understanding of the long-term impacts of nanomaterials. Long-term research is therefore required to examine the potential for resistance development over time. They enable researchers to examine genetic alterations, microbial adaptation, and the generation of resistant clones. Researchers can acquire a more accurate assessment of the potential limitations and effectiveness of nanomaterials by extending the duration of tests. Given the limits of short-term trials, it is crucial that future studies include longer testing periods to better understand the dynamics of microbial resistance and the possibility for the formation of resistant clones in the presence of nanomaterials [43].

Challenges related to human health are increasing in the growing world. The latest approaches are needed to combat them both sufficiently and adequately. Nanotechnology and its role in antibiotic resistance are powerful tools. This advancement is a promising tool not only for eliminating environmental pollution, but also for reducing antibiotic resistance. Therefore, to attain a stable, global environment, living organisms at the microscopic level should be dealt with first to provide a satisfactory macroscopic stability. Nano-wars have been proven as a treatment of bacteria, such as in human treatment through antibiotics. This approach has the potential against bacterial resistance with more nano-targets and environmental cleaning approaches. Moreover, deploying new theories and methodologies that take evolutionary ecology into account can bring beneficial insights and potential solutions. The concept of evolutionary traps, as outlined in Williams PD’s paper “Darwinian interventions: taming pathogens through evolutionary ecology,” provides an innovative viewpoint. Evolutionary traps seek to exploit trade-offs within microbial populations, making it harder for them to overcome several antagonistic challenges, such as drugs or interventions. It may be possible to establish conditions that lead to the selection of less resistant or more sensitive microbial strains by intentionally developing treatments that target these trade-offs, such as combining antibiotics, ionic/nano metals, or phages. This method emphasizes the significance of understanding these microbial evolutionary processes and their responses to various interventions. It may be possible to build more effective and long-lasting resistance-fighting techniques by applying evolutionary concepts. It is also critical to continue investigating and exploring such novel techniques to address the growing problem of microbial resistance. Evolutionary ecology-based therapies have the potential to provide new insights and influence the development of future interventions that can better manage resistance and improve treatment results [44], for example, in the case of antibiotic resistance in bacteria. Traditional approaches involve the use of antibiotics to kill off susceptible bacteria, but this often leads to the survival and proliferation of resistant strains. However, by applying evolutionary ecology-based therapies, researchers can leverage these evolutionary principles to strategically deploy antibiotics in a way that creates evolutionary traps for resistant bacteria. By administering antibiotics at specific doses and intervals, it becomes possible to impose selective pressures that favor the extinction of resistant strains while allowing susceptible ones to thrive. Furthermore, phages, which are viruses that infect bacteria, can also be utilized in an evolutionary ecology-based approach. Phages have the ability to evolve alongside bacteria, continuously adapting to infect and kill their hosts. By better understanding the coevolutionary dynamics between the phages and bacteria, researchers can design phage-based interventions that exploit evolutionary principles to combat antimicrobial resistance effectively. Similarly, nanomaterials can be employed in an evolutionary ecology-based therapy. Nanoparticles can interact with bacteria in various ways, such as disrupting their cell membranes or interfering with their metabolic processes. By understanding the evolutionary responses of bacteria to nanomaterials, scientists can engineer nanoparticles that act as evolutionary traps. These traps can impose strong selective pressures on bacteria, leading to their extinction or reduced virulence. In summary, evolutionary ecology-based therapies have the potential to revolutionize the field of antimicrobial interventions. By harnessing the power of evolutionary theory and applying it to the deployment of antibiotics, phages, or nanomaterials, we can develop innovative strategies to manage resistance, enhance treatment outcomes, and pave the way for sustainable antimicrobial approaches.

## Figures and Tables

**Table 1:** Examples of applied nanomaterials to combat drug resistance.

Nanomaterials	Applications	References
Lipid-based nanoparticles	Targeted delivery of antibiotics to combat bacterial resistance	[29]
Metal-based nanoparticles	Enhancing the efficacy of antimicrobial agents against resistant pathogens	[30]
Polymeric nanoparticles	Overcoming multidrug resistance in cancer cells through combination therapy	[31]
Mesoporous silica nanoparticles	Controlled release of antimicrobial agents to overcome resistance in bacteria	[32]
Quantum dots	Imaging and monitoring drug-resistant cancer cells for personalized medicine	[33]
Dendrimers	Delivery of gene-silencing agents to combat drug resistance in viral infections	[34]
Nanoemulsions	Targeted delivery of antimicrobial agents against resistant pathogens	[35]
Gold nanoparticles	Overcoming multidrug resistance in chemotherapy	[36]

## Data Availability

All the data are available in the current opinion paper.
